# Closing the Loop with Gates: A Scale-up-Gated Design–Build–Test–Learn Framework for Industrial Fermentation

**DOI:** 10.3390/microorganisms14081830

**Published:** 2026-08-19

**Authors:** Xiang He, Yanling Hu, Yao Zhu, Xinli Li, Kenan Wang, Liqing Dong, Xiaolong He, Yueqin Liu, Jianzhao Qi, Pengfei Jin

**Affiliations:** 1 Shaanxi Key Laboratory of Research and Utilization of Resource Plants on the Loess Plateau, Engineering Research Center of Microbial Resources Development and Green Recycling of the University of Shaanxi Province, College of Life Science, Yan’an University, Yan’an 716000, China; m18970421008_1@163.com (X.H.); 19161521970@163.com (Y.H.);; 2 Center of Edible Fungi, Northwest A&F University, Yangling, Xianyang 712100, China

**Keywords:** fermentation industry, synthetic biology, artificial intelligence, Design–Build–Test–Learn loop, scale-up constraints, industrial biomanufacturing

## Abstract

The global fermentation industry faces persistent bottlenecks in scaling laboratory innovations to industrial production, and the integration of synthetic biology (SynBio) and artificial intelligence (AI) within the Design–Build–Test–Learn (DBTL) loop has yielded inconsistent industrial outcomes. This review proposes that transformative impact requires a “scale-up-gated DBTL” framework, in which explicit decision gates constrain every iteration. At the Design phase, scale-down simulation data must inform genetic design choices. At the Test phase, downstream processing compatibility and industrial robustness metrics are enforced as non-negotiable evaluation criteria. At the Learn phase, techno-economic analysis (TEA) and life-cycle assessment (LCA) serve as the convergence criteria, replacing traditional titer plateaus. Through a qualitative cross-sectoral analysis of food, pharmaceutical, agricultural, and energy fermentation, the analysis reveals that workflows incorporating such constraints consistently bridge the valley of death, whereas unconstrained DBTL systematically converges on laboratory optima that are industrially unviable. Five strategic priorities are outlined—embedding TEA/LCA into DBTL, adopting scale-down simulation, building open fermentation data repositories, harmonizing regulatory frameworks, and fostering cross-disciplinary training—as prerequisites for progressing toward fully autonomous, scale-up-aware biomanufacturing.

## 1. Introduction

For millennia, fermentation existed as a craft-based empirical practice, spanning ancient Sumerian beer production to traditional bread and soy sauce manufacturing. The 20th century elevated it to a formal engineering discipline, driven by foundational advances in bioreactor design and recombinant genetic engineering. According to Grand View Research, the global fermentation sector constitutes a core pillar of the modern bioeconomy, with its total market value projected to exceed USD 501 billion by 2030 [1].

Nevertheless, the sector currently stands at a critical inflection point. Conventional development strategies—including random mutagenesis, empirical parameter optimization, and heuristic scale-up protocols—have yielded diminishing marginal returns [2]. Strains engineered for optimal performance in shake-flask cultures frequently exhibit impaired productivity or viability when scaled to 100 m^3^ industrial bioreactors [3,4,5]. Traditional process control strategies, which rely exclusively on bulk parameters such as temperature and pH, lack the capacity to directly modulate intracellular metabolic fluxes [6]. Downstream processing (DSP) continues to account for 20–60% of overall manufacturing costs [7,8]. While the sector has achieved robust control over microbial cultivation at scale, it has yet to establish a framework for engineering microbial strains as predictable, reliable manufacturing platforms [9,10].

To characterize the current landscape, we surveyed review articles from the past decade (2015–2025) indexed in Web of Science and PubMed. From this corpus, three representative reviews were selected to illustrate the dominant, tool-centric orientation of the literature—covering genome editing, adaptive laboratory evolution, and chassis engineering, respectively [11,12,13]. A unified, integrative perspective that explicitly connects these tools to industrial translation remains limited. Furthermore, the current body of literature remains largely descriptive in scope: synthetic biology (SynBio) and artificial intelligence (AI) are typically examined as discrete fields; the profound synergies that arise when both are integrated within a closed-loop engineering framework—and, crucially, constrained by manufacturability criteria—have not been systematically addressed [14]. This fragmentation provides empirical snapshots rather than a coherent development roadmap.

The Design–Build–Test–Learn (DBTL) cycle—wherein computational design informs genetic construction, variants are experimentally tested, and results are learned to refine subsequent iterations—has become the dominant operational paradigm in metabolic engineering. This review argues that the sector requires a fundamentally different approach to this cycle. The critical question is not whether to adopt DBTL, but what decision criteria govern each iteration. The authors argue that impactful DBTL implementations are distinguished by explicit scale-up constraints embedded as decision gates: scale-down simulation data must constrain genetic design choices at the Design phase; DSP compatibility and industrial robustness metrics must gate performance evaluation at the Test phase; and TEA/LCA must serve as convergence criteria—not titer plateau—at the Learn phase. This is term “scale-up-gated DBTL.” SynBio and AI remain essential tools, but both are subordinate to the gate logic. It is the gates, not the tools, that close the loop.

This framework directly addresses the three core bottlenecks: the genotype–phenotype gap, the limits of environmental process control, and the DSP cost burden. By embedding scale-up constraints at every pipeline phase, scale-up-gated DBTL systematically narrows the gap between laboratory feasibility and industrial manufacturability. The authors argue that the valley of death—the persistent failure to translate laboratory proof-of-concept into profitable production [15]—is largely the aggregate consequence of unconstrained DBTL iterations that optimize for laboratory metrics without gating for manufacturability. Bridging this gap demands a holistic understanding of interactions across biological, engineering, and economic scales [16]. This review contributes to a critical, forward-looking synthesis to guide both academic research and industrial planning over the coming decade.

## 2. Diagnosing the Bottlenecks: Why Unconstrained DBTL Fails at Industrial Translation

### 2.1. Strain Engineering: The Genotype–Industrial Phenotype Chasm

Strain engineering defines the theoretical upper bounds of fermentation performance, including maximum product yield, substrate utilization range, and end-product quality [12]. Two dominant strategies underpin modern strain development: CRISPR-based genome editing for precise, targeted genetic modifications, and adaptive laboratory evolution (ALE), which leverages natural selection to enhance complex, polygenic phenotypic traits [12,17]. Despite their well-documented successes at laboratory scale, a persistent disconnect remains between engineered genotype and industrial phenotype—defined as a strain’s actual performance under the heterogeneous, dynamic, and suboptimal conditions of production-scale bioreactors [18].

CRISPR-Cas9 and its derived systems enable targeted gene knockouts, genomic insertions, and pathway-level expression regulation with unprecedented precision and throughput. Under controlled laboratory conditions, these tools are routinely used to generate high-yield strains by ablating byproduct-biosynthetic pathways or overexpressing rate-limiting enzymes in core metabolic pathways [17]. However, laboratory-scale validation seldom recapitulates the physicochemical environment of industrial bioreactors. Shake-flask cultures and small-scale benchtop bioreactors fail to reproduce the spatial gradients of dissolved oxygen, pH, substrate concentration, and shear stress that are inherent to large-volume vessels. As a result, strains engineered to achieve maximum titer under homogeneous, well-controlled conditions frequently display diminished robustness, compromised stress tolerance, or unanticipated metabolic burden upon scale-up [19,20].

Unlike rational genome editing, ALE does not rely on prior knowledge of the genetic basis underlying target traits. By imposing sustained selective pressure—such as elevated temperature, low pH, or high concentrations of inhibitory products—ALE drives the accumulation of beneficial mutations that improve target phenotypic traits [12,21]. Notably, as a non-genetically modified organism (non-GMO) approach, strains developed via ALE may offer regulatory advantages in certain jurisdictions, although their classification varies across regulatory frameworks [22]. Nevertheless, ALE is inherently time-intensive, typically requiring hundreds to thousands of generations of cultivation; additionally, the causal mutations underlying evolved traits are often polygenic and challenging to identify and validate [12,23]. Furthermore, evolved phenotypes are frequently associated with fitness trade-offs; for example, enhanced stress tolerance may come at the expense of maximum specific growth rate.

Why, then, do so many engineered strains exhibit diminished performance at industrial scale? Three interconnected, mechanistic factors are consistently underestimated in laboratory strain development. First, metabolic burden: the expression of heterologous biosynthetic pathways diverts cellular precursors (e.g., amino acids, nucleotides, and cofactors), ATP, and reducing equivalents away from native growth and maintenance metabolism. This metabolic redistribution often induces cellular stress responses, which in turn reduce biosynthetic productivity or create selective pressure that favors non-producing revertant mutants [24]. Second, off-target effects: even highly precise CRISPR-mediated edits can perturb global transcriptional networks, imposing fitness disadvantages that become pronounced under the fluctuating environmental conditions of industrial bioreactors [25]. Third, genetic instability: during extended fermentation cycles, non-producing mutants or plasmid-free variants can outcompete and overtake the engineered production population [26]. High cell densities and prolonged cultivation times are well recognized to drive reductions in productivity through genetic and expression instability [27]. This effect is particularly pronounced in fed-batch or continuous cultivation modes, where there is no direct selective pressure to maintain product biosynthesis.

Diagnosis from the DBTL perspective. The persistent genotype–industrial phenotype gap is not primarily a failure of strain engineering tools—CRISPR and ALE are operationally mature in terms of throughput and laboratory-scale cost, though their predictive power for industrial-scale performance remains limited—but a failure of the DBTL workflow within which these tools are deployed. In standard practice, the Design phase relies almost exclusively on performance data obtained under homogeneous, well-controlled shake-flask or benchtop conditions. Scale-down simulation data—capturing the dissolved oxygen gradients, pH fluctuations, substrate heterogeneity, and shear forces characteristic of industrial bioreactors—are rarely, if ever, used as Design phase inputs. Consequently, strains are built for laboratory fitness, not industrial robustness. The metabolic burden, off-target effects, and genetic instability documented above are thus predictable consequences of a Design phase constraint deficit: the DBTL loop, as commonly executed, lacks a decision gate that demands demonstration of industrial-condition performance before a strain proceeds to Build. Without such a gate, DBTL does not solve the scale-up problem—it merely accelerates the rate at which laboratory-optimized strains encounter industrial failure.

### 2.2. Process Control: The Limits of Environmental Regulation

Fermentation process control has historically relied on the regulation of a limited set of measurable bulk physicochemical parameters: temperature, pH, dissolved oxygen, agitation speed, and substrate feeding rate [28]. Distributed control systems (DCS) are widely deployed to monitor these variables and implement adjustments via closed feedback loops [29]. This strategy delivers adequate performance when microbial growth kinetics and product biosynthesis exhibit predictable, consistent correlations with bulk environmental conditions. Nevertheless, this framework is constrained by a fundamental limitation: modulation of the extracellular environment does not exert direct control over intracellular metabolic states. Parameters such as temperature and pH act as indirect, global modulators of cellular metabolism, and their perturbation often elicits multiple, frequently counteracting, physiological responses [6]. An operational adjustment that improves product yield may concurrently reduce strain viability or elevate byproduct accumulation, and such inherent trade-offs cannot be fully resolved through bulk environmental regulation alone.

The demand for direct, targeted regulation of intracellular metabolic processes has intensified alongside the growing adoption of highly engineered strains and the production of structurally complex fermentation products. Conventional induction systems (e.g., IPTG, lactose) offer only coarse temporal control and are often associated with pleiotropic physiological effects [30,31]. Furthermore, in large-scale bioreactors, spatial gradients in substrate concentration and dissolved oxygen generate heterogeneous microenvironments [32]. Cells distributed across these zones experience transient, fluctuating environmental conditions, which give rise to population-level metabolic heterogeneity and a net reduction in overall process productivity [33].

This limitation—the inability to exert direct control over intracellular metabolic states—is not attributable solely to insufficient sensor performance. Even with high-fidelity, real-time monitoring of pH, temperature, and dissolved oxygen, intracellular concentrations of pathway intermediates, the redox status of key cofactors, and the expression levels of heterologous biosynthetic enzymes cannot be directly accessed within conventional control architectures. These intracellular variables represent the direct determinants of biosynthetic productivity, yet they remain invisible to distributed control systems that operate exclusively on extracellular physicochemical parameters.

Additionally, fermentation processes exhibit strongly nonlinear, time-varying dynamic behavior [34]. Proportional–integral–derivative (PID) controllers calibrated for the early exponential growth phase often exhibit degraded performance during stationary phase or following a metabolic shift [35]. While adaptive control strategies have been developed, their implementation depends on mechanistic models that accurately describe the relationship between extracellular environmental inputs and cellular phenotypic outputs. Such models are challenging to derive from first principles owing to the inherent complexity of microbial metabolic networks.

Diagnosis from the DBTL perspective. The inability to modulate intracellular metabolic states via bulk environmental control is not solely a sensor or actuator limitation—it is also a Test-phase constraint deficit within the prevailing DBTL paradigm [36]. The Test phase, as conventionally configured, evaluates strain and process variants against a narrow set of laboratory performance metrics: titer, specific yield, and volumetric productivity, typically measured under idealized conditions. Critically, downstream processing (DSP) compatibility indicators—including product localization (extracellular secretion vs. intracellular accumulation), the complexity of the impurity profile generated by the production strain, and broth rheological properties that affect membrane filtration and chromatography efficiency—are excluded from the Test matrix [37]. Consequently, the Learn phase receives a systematically biased dataset: it is trained to recognize “high performance” as “high titer,” without any signal regarding the purification cost that will be incurred to recover that titer at industrial scale. The resulting optimization trajectory maximizes bioreactor output at the expense of downstream economics unconstrained DBTL, in this context, does not resolve the process control bottleneck; it refines control toward the wrong objective function.

### 2.3. Downstream Processing: The Hidden Cost Barrier

Downstream separation and purification (DSP) represent one of the most underappreciated bottlenecks in industrial fermentation process development. While upstream strain engineering and bioreactor process optimization remain the primary focus of research and development efforts, DSP typically accounts for 20–60% of total manufacturing costs; for high-purity pharmaceutical products including biopharmaceuticals and monoclonal antibodies, this proportion can rise to 50–80% [7,38]. Fermentation broths are highly complex multiphase systems consisting of microbial biomass, residual media components, metabolic byproducts, and the target product, which is often present at relatively low concentrations [39]. Recovery and purification of the target product to meet regulatory purity specifications requires a sequence of unit operations—including centrifugation, filtration, chromatography, crystallization, and drying—each of which incurs additional capital expenditure (CAPEX) and operating expenditure (OPEX) [40]. Consequently, a fermentation process that achieves record-high product titers at the bioreactor stage may still fail to achieve economic viability if downstream cost implications are not addressed from the earliest stages of process design.

The economic constraints of DSP impose a fundamental trade-off between product commercial value and purification expenditure. For low-value bulk commodities such as bioethanol and organic acids, downstream processes must be highly cost-effective and operationally robust; high-cost unit operations such as preparative chromatography or advanced membrane systems are economically unfeasible [41]. For high-value products including pharmaceutical ingredients and rare sugars, higher purification costs are commercially viable, but the consequences of process failure—such as product recalls or regulatory non-compliance—are substantial. This dichotomy drives technology selection across industrial sectors but does not alleviate the underlying pressure to reduce downstream costs.

Recent technological developments have expanded the available DSP toolbox, but each emerging approach carries associated economic trade-offs. Membrane separation technologies have achieved enhanced ion selectivity using novel membrane materials, yet persistent challenges including membrane fouling and the inherent selectivity–permeability trade-off remain unaddressed at industrial scale [42,43]. Magnetic separation, which employs functionalized magnetic particles, presents an alternative to conventional packed-bed chromatography, but the high costs of consumable particles and dedicated processing equipment require thorough techno-economic assessment [44]. Integrated continuous downstream processing, in which multiple unit operations are coupled into a single continuous workflow, has demonstrated promising performance at laboratory scale; however, such systems require substantial upfront capital investment and introduce additional operational complexity [45].

A critical limitation of current development practices is that DSP is frequently treated as a secondary, post hoc consideration. Many strain development programs focus exclusively on improving bioreactor titer, operating under the assumption that downstream purification challenges can be resolved at a later development stage. This assumption proves problematic when engineered strains generate novel, hard-to-separate byproducts, or when intracellular product localization necessitates additional cell disruption unit operations. The resulting misalignment between upstream production performance and downstream process feasibility yields processes that function reliably at laboratory scale but cannot be scaled to industrial production in an economically sustainable manner [2].

Diagnosis from the DBTL perspective. The disproportionate contribution of DSP to overall manufacturing costs is not merely a downstream technical challenge—it is a structural defect in how the DBTL loop allocates decision authority across its phases. When DSP is treated as a post hoc consideration, strain and process variants are allowed to proceed through multiple Design–Build–Test iterations without ever facing a DSP-related decision gate. This means that variants generating difficult-to-separate byproducts, accumulating product intracellularly, or producing high-viscosity broths are not identified as problematic until substantial resources have already been invested in their development. In a scale-up-gated DBTL framework, DSP compatibility must function as a Test-phase gate: any variant that fails to meet pre-defined DSP cost thresholds should return to the Build phase for re-engineering of product localization, byproduct profiles, or other purification-relevant traits, and must not advance to the next Design cycle. These cost thresholds can be assessed using scaled-down purification mimics or predictive chromatography models. The former use miniaturized devices (e.g., microscale centrifugation, membrane filtration, or chromatography columns) to replicate manufacturing-scale hydrodynamics at milliliter volumes, enabling early prediction of large-scale performance with minimal material. The latter—encompassing mechanistic (physicochemical) and data-driven (e.g., QSAR/QSPR) approaches—simulate separations in silico to accurately predict optimal resin, pH, conductivity, and gradient conditions, thereby greatly reducing experimental screening. Without this gate, DBTL can efficiently converge on a strain that achieves record titers and simultaneously renders the overall process economically nonviable—an outcome that is worse than failure, because it consumes resources that could have been directed toward manufacturable solutions.

### 2.4. Cross-Sectoral Synthesis: Differentiated Constraint Sensitivities Across Industrial Sectors

While Section 2.1, Section 2.2 and Section 2.3 dissected the technical roots of the three core bottlenecks, the diagnostic lens of scale-up-gated DBTL reveals a deeper pattern: each fermentation sector suffers from a distinct constraint deficit within the DBTL loop, and the bottleneck that dominates industrial outcomes is sector-specific.

Food fermentation is most sensitive to a Design gate deficit. Consumer GMO aversion and the primacy of sensory quality impose non-negotiable market constraints external to fermentation science [46]. Conventional DBTL deploys CRISPR to maximize titer, but the Design phase omits consumer-acceptability and sensory metrics as decision criteria. High-titer-engineered strains are consequently rejected at commercialization due to off-flavor byproducts or GMO status [47,48]. Non-GMO approaches such as ALE are available but underprioritized because their lower throughput is penalized by unconstrained workflows that reward speed over market fitness.

Fermentation for pharmaceutical processes is most sensitive to a Test gate deficit—specifically, the omission of DSP compatibility from the Test matrix. DSP accounts for 50–80% of OPEX in this sector, and cGMP/QbD requirements make post hoc process changes prohibitively expensive [49]. Yet conventional DBTL evaluates variants almost exclusively against titer and yield under idealized conditions, excluding DSP-relevant indicators—product localization, impurity profiles, broth rheology—from the Test matrix. The Learn phase therefore converges on “high titer” while the purification cost required to recover that titer at scale remains invisible to the optimization algorithm.

Fermentation processes applied in agriculture are most sensitive to a Learn gate deficit—the absence of TEA as a convergence criterion. Products are priced at a few USD per kilogram, and grower adoption is driven by ROI rather than environmental claims [50]. Unconstrained DBTL allows variants to be declared “optimized” without confronting this OPEX ceiling. Processes that clear Design and Test on technical merit fail at commercialization because TEA was performed retrospectively, if at all. Solid-state fermentation and low-cost substrate strategies remain underprioritized despite being critical for sector viability [51].

Energy and environmental fermentation is most sensitive to a Design gate deficit of a different kind—the failure to incorporate industrial-condition robustness as a Design input. Lignocellulosic inhibitors and gas fermentation toxicity impose selection pressures absent from laboratory batch cultures [52]. Design phases relying on mild conditions fail to filter out strains destined for robustness collapse upon scale-up. The success of *C. autoethanogenum* [53] can be retrospectively understood as an implicit practice of scale-up-gated DBTL: a non-model chassis was selected because conventional hosts failed industrial-condition robustness criteria that functioned, in effect, as a Design gate.

This cross-sectoral analysis yields a practical implication: there is no universal scale-up-gated DBTL protocol. The constraint that must be embedded as a decision gate—and the phase at which it is enforced—depends on the economic, regulatory, and market logic of the target sector. What unifies these divergent cases is a single principle: a DBTL loop lacking sector-appropriate decision gates is structurally incapable of bridging the valley of death.

Beyond these sector-specific gate deficits, a more fundamental distinction merits emphasis: the roles of scale-down simulation and TEA/LCA are complementary but distinct in the DBTL framework. Scale-down simulation is the primary bioengineering tool for translating biological and engineering phenomena into process design decisions [3,5]. Miniature bioreactor systems that replicate the spatial gradients of dissolved oxygen, pH, and substrate concentration, as well as hydrodynamic stresses and mass transfer limitations inherent to large-volume vessels, enable researchers to predict strain performance under industrial conditions before committing to pilot-scale investment [32]. These platforms capture the heterogeneous microenvironments that drive population-level metabolic heterogeneity and productivity losses—phenomena that cannot be reproduced in shake-flask cultures. TEA/LCA, by contrast, evaluates the economic and environmental outcomes of the resulting process design after bioengineering constraints have been addressed. In the scale-up-gated framework, scale-down simulation thus informs Design- and Test-phase decisions (what strain and process parameters are biologically and physically viable at scale), while TEA/LCA serves as the Learn phase convergence criterion (whether that viable process is economically and environmentally sustainable). This distinction ensures that bioengineering design decisions and economic viability assessments are addressed by their respective appropriate tools, rather than conflated.

## 3. The DBTL Solution: Closing the Loop Between Design and Industrial Reality

### 3.1. The DBTL Loop as a Scale-up-Gated Framework

The DBTL iterative cycle comprises four phases: Design generates candidate strategies via computational models and empirical knowledge; Build implements these designs as physical variants using SynBio and process engineering tools; Test quantifies variant performance under relevant conditions; and Learn analyzes experimental datasets—frequently via machine learning—to refine predictive models and inform subsequent Design cycles [54,55,56]. When integrated with high-throughput automation, this cycle can compress development timelines from years to months. However, cycle speed alone does not determine industrial outcomes.

**Figure 1:**
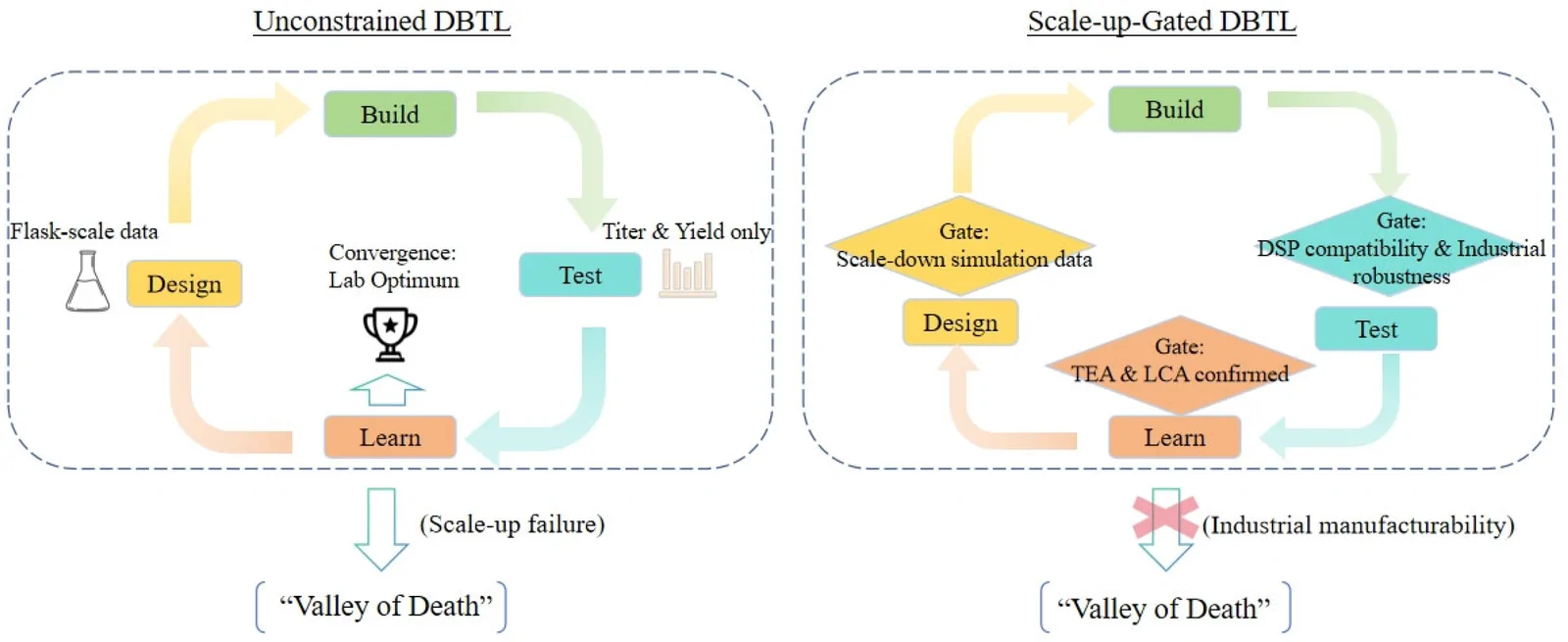
Contrasting the unconstrained and scale-up-gated DBTL paradigms.

It is argued here that the defining feature distinguishing impactful from ineffective DBTL implementations is the explicit incorporation of scale-up constraints at specific decision gates within the cycle. Without such gates, DBTL efficiently converges on solutions optimized for laboratory conditions—solutions that predictably fail upon scale-up. The alternative framework is termed the alternative scale-up-gated DBTL, in which three decision gates govern each iteration (Figure 1):

Design gate: Genetic design choices—promoter strength, copy number, growth-coupling strategy, chassis selection—must be constrained by scale-down simulation data that capture the spatial gradients, shear forces, and substrate heterogeneity of industrial bioreactors. A strain designed without this gate is optimized for shake-flask homogeneity, not bioreactor reality.

Test gate: Performance evaluation must extend beyond titer, yield, and productivity to include (i) DSP compatibility metrics—product localization, impurity profiles, broth rheology—and (ii) industrial robustness indicators—genetic stability under extended fed-batch, tolerance to transient oxygen or pH excursions. These are not optional “downstream considerations” but co-equal Test-phase criteria. Variants failing DSP or robustness thresholds do not advance.

Learn gate: Convergence is not declared when titer plateaus, but when TEA and LCA confirm that the combined upstream–downstream process meets pre-defined economic and sustainability thresholds. If an iteration improves titer but degrades overall process economics—for example, by increasing purification costs—the loop must continue. The Learn gate enforces the principle that laboratory performance is necessary but insufficient for industrial viability.

The Build phase, while not constituting an independent directional decision gate, executes a critical Build-internal quality control function that directly addresses the failure modes identified in Section 2.1. Before any variant proceeds to the Test phase, the fidelity of its construction must be proactively verified. This includes sequencing-based confirmation of intended edits, systematic screening for off-target CRISPR lesions, quantification of transformation efficiency, and early assessment of construct stability under selective pressure. These checks ensure that genetic instability—a primary driver of the genotype–industrial phenotype chasm—is detected and rectified within the Build phase itself, rather than being misattributed to scale-up failure at a later stage. Failure to pass Build-internal checks triggers a mandatory return to Build for re-engineering, functionally serving as a quality filter that conserves Test-phase resources for variants with verified genetic integrity. In this sense, Build operates as the execution arm of the Design gate’s decision: it translates gated design specifications into physical constructs and validates that the translation is faithful.

Within this framework, SynBio and AI serve distinct but subordinate roles. SynBio provides the experimental toolkit for constructing variants that satisfy the Design gate criteria—enabling precise genome editing, pathway assembly, and dynamic metabolic regulation [57,58]. AI accelerates the Test–Learn transition by identifying predictive patterns in high-dimensional datasets that human analysis cannot resolve [56,59]. Both are essential. But it is the gates—not the tools—that close the loop. A DBTL workflow equipped with state-of-the-art SynBio and AI, yet lacking these decision gates, remains an unconstrained optimization engine: faster, but directionally unimproved.

The applicability of this gated framework extends across the fermentation value chain—from strain engineering to bioreactor process optimization to DSP workflow design—precisely because the gate criteria, not the specific tools or phases, define its logic. This is what distinguishes scale-up-gated DBTL from generic invocations of the DBTL acronym.

### 3.2. Design and Build: SynBio as the Blueprint and Construction Toolkit

SynBio provides a systematic approach to bridging the genotype–industrial phenotype chasm (detailed in Section 2.1), by delivering a suite of tools that enable strain design and construction with industrial robustness as a core design criterion, rather than a post hoc consideration.

The Design phase initiates with definition of a target biosynthetic molecule and selection of a suitable microbial chassis. For well-characterized products, genome-scale metabolic models predict optimal biosynthetic pathway configurations and flux distributions. For novel compounds, de novo pathway design can circumvent native metabolic constraints that limit production efficiency. The following examples illustrate the current technical reach of SynBio in the Design and Build phases—demonstrating what is now achievable in terms of pathway design, heterologous expression, and regulatory control, rather than validating the gate-passing outcomes of the scale-up-gated framework. Guo et al. engineered a non-natural biosynthetic pathway in *Escherichia coli* for psilocybin production, successfully bypassing a native rate-limiting catalytic step [60]. Wu et al. constructed an artificial metabolic pathway for the production of branched β,γ-diols [61]. Chassis selection has also expanded beyond conventional model organisms such as *E. coli* and yeast, to include non-model microorganisms with unique native metabolic capabilities. *Bacillus subtilis*, generally recognized as a safe (GRAS) organism, has been engineered for industrial-scale production of lacto-N-tetraose [62], while *Serratia marcescens* has been harnessed as a platform for terpenoid biosynthesis [63].

The Build phase translates in silico design schemes into physical biological constructs. CRISPR-Cas9 and its derivative systems (CRISPRi, CRISPRa) enable marker-free, multiplexed genome editing across multiple genomic loci. Build-stage workflows also incorporate synthetic genetic circuits—including tailored promoters, terminators, and riboswitches—to enable dynamic, condition-dependent metabolic regulation. For example, in the context of antibiotic biosynthesis, Cai et al. inactivated a native transcriptional repressor in a production strain, achieving a 55% increase in lincomycin A titer [64].

Crucially, the Design and Build phases within the scale-up-gated DBTL framework go beyond these standalone technical demonstrations by explicitly integrating industrial robustness criteria into early-stage development. Rather than optimizing exclusively for maximum titer under shake-flask conditions, design teams leverage scale-down simulation data to select for traits including environmental stress tolerance, long-term genetic stability, and reduced metabolic burden. Strategies such as growth-coupled product biosynthesis and genetic firewall systems (e.g., toxin–antitoxin modules) are embedded into strain designs from the outset. The iterative structure of the loop enables continuous refinement: each Build–Test cycle identifies unanticipated failure modes, which directly inform design improvements for subsequent cycles. This systematic, gate-governed iterative approach distinguishes scale-up-gated DBTL from conventional single-cycle rational strain engineering that prioritizes laboratory titer above all else.

### 3.3. Test and Learn: AI as the Brain of the Loop

Where SynBio provides the experimental toolkit for physical construct generation, AI delivers the analytical capacity to extract actionable knowledge and guide iterative optimization—directly addressing the limitations of conventional environmental process control (detailed in Section 2.2) by enabling predictive and real-time modulation of intracellular metabolic states.

The Test phase generates large-volume, high-dimensional datasets spanning high-throughput strain variant screening, continuous process sensor time series, and whole-cell biosensor readouts. Manual data analysis cannot match the throughput and complexity of these datasets. Machine learning models, including neural networks and support vector machines, are well suited to identifying underlying patterns within noisy, nonlinear fermentation datasets, capturing subtle multivariate interactions that evade manual expert analysis [65].

The Learn phase moves beyond descriptive data analysis to generate predictive and prescriptive insights. Predictive models trained on historical process datasets can forecast the performance of novel strains or operating conditions, reducing the need for costly, low-throughput experimental trials. Chen et al. integrated CRISPR library screening with AI-driven predictive modeling to identify key genetic targets for high-performance production strains [66]. Hybrid artificial neural network–genetic algorithm (ANN–GA) models have been applied to optimize temperature, pH, and aeration parameters, increasing ethanol and xylitol yields by 22.3% and 13.3%, respectively [67].

AI further enables molecular-level process control—the direct modulation of intracellular metabolism via biosensors, quorum sensing systems, and regulatory non-coding RNAs [68]. In contrast to conventional distributed control systems (DCS), which act exclusively on the extracellular environment, molecular control interfaces directly with intracellular regulatory networks. AI algorithms learn the functional mapping between molecular input signals (e.g., inducer concentrations, light stimuli) and metabolic output fluxes, then generate optimal control policies that adapt dynamically in real time.

The digital twin—a dynamic, real-time virtual replica of a bioreactor and its microbial population—represents the most advanced integrated AI application in industrial fermentation [69]. Digital twin platforms integrate real-time process sensor data, first-principle mechanistic models, and machine learning algorithms to simulate current process states and forecast dynamic performance trajectories. These tools enable operators to simulate parameter adjustments, detect process deviations prior to failure events, and optimize production schedules without disrupting ongoing operations [70,71].

Nevertheless, AI is not a universal solution. Its performance is contingent on the quality and volume of training data, and industrial fermentation datasets are often limited in scale and confounded by process noise [65]. Trained models frequently exhibit poor generalizability across distinct production strains or process configurations [16]. Model interpretability also remains a key barrier to regulatory adoption, as health authorities such as the FDA and EMA maintain a cautious approach to black-box model deployment in cGMP manufacturing. These limitations are well-documented, and active research efforts are addressing them through advances in transfer learning, explainable artificial intelligence (XAI), and cross-industry data-sharing consortia.

**Figure 2:**
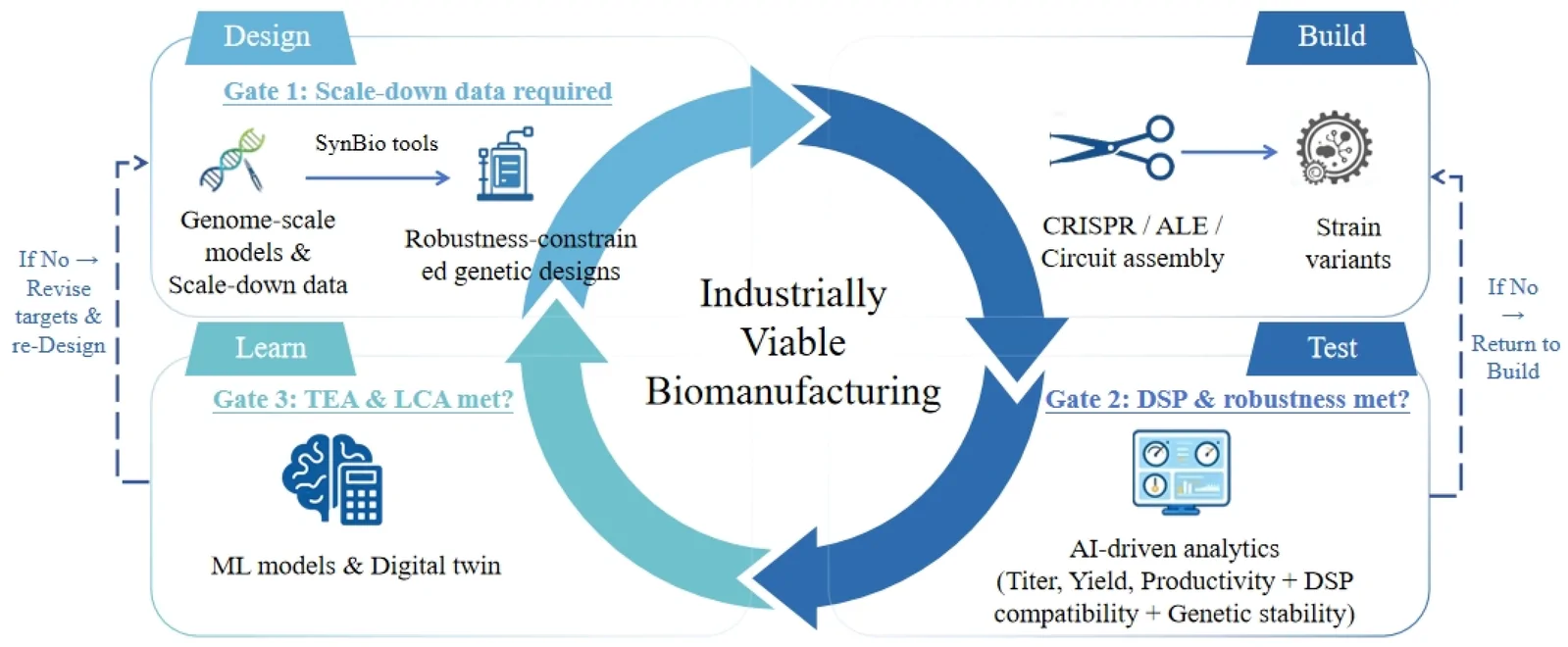
Detailed architecture of the scale-up-gated DBTL framework for industrial biomanufacturing.

The full architecture of the scale-up-gated DBTL framework, integrating the SynBio and AI tools discussed in Section 3.2 and Section 3.3 under the governance of three decision gates, is illustrated in Figure 2.

### 3.4. Testing the Argument: Evidence from Integrated SynBio–AI Workflows

The scale-up-gated DBTL framework is not merely a theoretical construct. Integrated SynBio–AI platforms have already demonstrated industrial successes that, when examined through the gate logic proposed here, reveal a consistent pattern: what distinguishes successful from failed translation is not the sophistication of tools deployed, but whether scale-up constraints functioned as effective decision gates. This section re-examines published case studies to test this claim—including both positive evidence (cases where gating was practiced, explicitly or implicitly) and the negative evidence of systematic failure patterns attributable to its absence.

Gas fermentation: implicit Design gating via chassis selection. Liew et al. successfully scaled acetone and isopropanol production using *Clostridium autoethanogenum* from pilot to full commercial production, achieving stable, carbon-negative operation [53]. The conventional interpretation frames this as a DBTL success. From a scale-up-gated perspective, the decisive factor was more specific: the Design phase implicitly enforced an industrial-robustness gate that conventional hosts could not satisfy. *C. autoethanogenum* was selected not because it was the most tractable chassis for genetic engineering—*E. coli* would have been far easier to manipulate—but because it natively tolerates the gas fermentation environment (CO, CO_2_, syngas toxicity) and sustains autotrophic metabolism under conditions that would impose lethal metabolic burden on heterologous pathways in model organisms. The Test phase further reinforced this gate: validation was conducted under pilot-scale industrial conditions, not merely in laboratory-scale idealized settings. Had a conventional unconstrained DBTL workflow been applied—designing pathways in *E. coli*, building high-titer laboratory strains, testing in shake-flasks, and learning toward ever-higher titer—the resulting strains would have collapsed upon first exposure to syngas. The Liew et al. case thus demonstrates that a Design gate enforcing industrial-condition robustness—even when practiced implicitly—can succeed where unconstrained optimization of an unsuitable chassis would fail.

Alternative protein production: Test gating via manufacturability constraints. Priyadharshini et al. reported an integrated DBTL platform for alternative protein production in which CRISPRi-mediated gene knockdown (Build) was coupled with high-throughput automated fermentation and LC–MS quantification (Test), followed by machine learning-driven prediction of optimal expression levels (Learn). Published results indicate yield improvements of up to 300%, alongside an 80% reduction in experimental trials [72]. The conventional narrative emphasizes AI-driven efficiency. From a scale-up-gated perspective, however, a more subtle and arguably more consequential feature of this platform deserves attention: the Test phase incorporated manufacturability indicators—including protein solubility, secretion efficiency, and impurity profiles—alongside titer. Without these DSP-relevant metrics embedded in the Test matrix, the AI component would have efficiently converged on expression configurations that maximize titer irrespective of purification cost. The 80% reduction in experimental trials, impressive as it is, would have been deployed in service of the wrong objective function. The platform’s success therefore illustrates the Test gate principle: when DSP compatibility is elevated to a co-equal evaluation criterion alongside titer, the Learn phase converges on globally economic optima rather than locally maximal titers.

The negative evidence: what the literature does not report. The academic literature contains hundreds of studies reporting record titers achieved in shake-flasks that never progress beyond pilot scale. These failures are rarely published—they constitute a “silent evidence” problem that systematically biases the published record toward laboratory success [2]. From the diagnostic perspective developed here, many of these translation failures are attributable not to the absence of DBTL, but to DBTL workflows that operated without scale-up constraints at the Design and Test gates [54]. Strains were designed for shake-flask homogeneity, not bioreactor heterogeneity. Performance was tested exclusively against titer, without DSP compatibility or industrial robustness metrics. Convergence was declared at the Learn phase on the basis of laboratory metrics alone. In each of these dimensions, the loop did close—but it closed on a laboratory optimum that was industrially unviable. This is the signature failure mode of unconstrained DBTL, and its prevalence across the fermentation literature underscores the practical urgency of the scale-up-gated framework.

Scope and limitations. These case studies demonstrate that gating—whether explicitly formalized or implicitly practiced—is a necessary condition for successful industrial translation. They do not, however, demonstrate sufficiency. Even a fully gated DBTL workflow faces barriers—CAPEX requirements, regulatory heterogeneity, workforce skill gaps—that lie outside the loop’s technical logic. These barriers are addressed in Section 4.

## 4. From Lab to Factory: Industrialization Bottlenecks and Strategic Pathways

### 4.1. Global Competitive Landscape: Regional Differentiation of Core Capabilities

The global fermentation industry is not a homogeneous entity; distinct regional strengths, regulatory frameworks, and market structures shape a highly differentiated competitive landscape.

The United States leads in two high-value segments: synthetic biology (SynBio) startup innovation and biopharmaceutical fermentation manufacturing [73]. The U.S. SynBio market was valued at approximately $15 billion in 2024, with North America holding the largest global market share of 41.8–42.1% [74]. Similarly, North America accounted for approximately 41% of the global biopharmaceutical fermentation systems market and 54.8% of estimated world pharmaceutical sales [75,76]. A dense ecosystem of venture capital, top-tier research universities, and contract development and manufacturing organizations (CDMOs) has created a dynamic innovation environment in which novel platform technologies—including AI-driven strain design and automated DBTL biofoundries—emerge and scale rapidly [77]. Companies developing precision fermentation platforms for alternative proteins and gas fermentation for commodity chemicals have completed industrial pilot-scale validation at unprecedented rates [53]. However, US leadership is less prominent in traditional food fermentation and large-scale commodity manufacturing, where production capacity has largely migrated to Asian markets—which collectively account for approximately 40% of the global microbial fermentation technology market [78].

Europe retains long-standing strengths in food and pharmaceutical fermentation, supported by a regulatory framework that prioritizes sustainability and circular bioeconomy principles [79]. European firms dominate market segments such as starter cultures, probiotics, and specialty industrial enzymes. Stringent EU environmental regulations have also driven innovation in waste-to-value fermentation and energy-efficient bioprocess design. Nevertheless, the adoption of genetically modified organisms (GMOs) in food applications has lagged in the region, constrained by public skepticism and complex regulatory approval pathways, which limits the deployment of SynBio tools in this sector [80].

China has achieved dominant production scale across multiple fermentation segments and ranks among the world’s largest producers of commodities including amino acids, organic acids, antibiotics, and bioethanol, a position driven by low manufacturing costs and robust policy support for the biotechnology sector [81]. However, the industry faces well-documented structural challenges: reliance on imported high-value production strains and industrial enzymes, a fragmented landscape of small-scale enterprises characterized by high product homogeneity and low-value addition, and rising environmental compliance costs that exert growing pressure on low-margin producers. Core technological gaps persist in high-value segments such as food-grade starter cultures, advanced biopharmaceutical expression systems, and high-efficiency downstream processing equipment [82].

Other regional players—including Japan, South Korea, and India—occupy specialized niche market positions. Japan excels in high-value fermentation products (e.g., amino acids, nucleotides) and advanced process automation technologies. South Korea has made substantial strategic investments in red biotechnology focused on biopharmaceutical manufacturing. India leverages low labor costs and a rapidly expanding bioeconomy, but faces constraints related to industrial infrastructure gaps and batch-to-batch process consistency.

A key observation is that no single region dominates all dimensions of the fermentation value chain: the United States leads in early-stage technological innovation, Europe in sustainable food fermentation and regulatory frameworks for natural products, and China in large-scale manufacturing capacity and cost competitiveness. The coming decade is expected to bring intensified global competition, alongside growing opportunities for cross-border collaboration—for example, US-developed production strains manufactured at scale in Asian facilities, or European process engineering expertise applied to Chinese production capacity.

### 4.2. The “Valley of Death” in Fermentation Industrialization

Across all regional markets, the most persistent and understudied commercialization bottleneck is the widely documented “valley of death”: the critical gap between successful pilot-scale demonstration and profitable full-scale industrial production [83]. For every novel fermentation process that successfully scales to 10,000 L bioreactors, dozens fail to progress beyond the benchtop (10 L) to pilot (1000 L) transition. The underlying barriers fall into four interconnected categories.

From the diagnostic perspective developed in this review, these barriers are not independent obstacles that happen to co-occur. Rather, they are the aggregate consequence of unconstrained DBTL iterations—workflows that optimize for laboratory performance metrics without gating for industrial manufacturability. When strains are designed without scale-down simulation input, tested without DSP compatibility metrics, and declared converged without TEA confirmation, the resulting process carries predictable vulnerabilities—to scale-up failure, to cost overrun, and to regulatory delay—that manifest as the valley of death. The four barrier categories below can thus be understood as the downstream symptoms of a common upstream deficit: the absence of scale-up decision gates in the DBTL loop.

First, high capital expenditure (CAPEX) constitutes a major entry barrier. Commercial-scale fermentation facilities require integrated infrastructure beyond core bioreactors—including media preparation, downstream purification trains, wastewater treatment, and quality control laboratories—with total construction costs for a greenfield plant typically reaching nine figures (USD) [84,85]. For early-stage startups and academic spin-offs, securing this level of capital is exceptionally challenging, particularly as investors demand prior de-risking that can only be provided by a fully operational pilot facility—a classic ‘chicken-and-egg’ dilemma.

Second, high operating expenditure (OPEX) undermines long-term commercial viability. Industrial fermentation is energy-intensive, with substantial power demand for sterilization, aeration, agitation, and downstream purification [83]. Water consumption is also significant, and wastewater treatment costs can be prohibitive, especially for processes generating recalcitrant byproducts or volatile organic compounds [86]. Furthermore, strain performance that appears favorable in shake-flask culture frequently deteriorates at industrial scale due to unanticipated metabolic burden, genetic instability, or sensitivity to spatial concentration gradients and shear forces in large-volume vessels [4]. These scale-related performance losses result in reduced product titers, extended batch cycle times, or elevated contamination rates—all of which erode or eliminate process profitability [26].

Third, regulatory compliance and process validation costs represent a substantial barrier, particularly for pharmaceutical and food fermentation products [45]. A modification to the host strain background or even the culture medium composition can trigger mandatory comparability assessments that cost millions of dollars and require several years to complete [87]. Regulatory frameworks for SynBio-derived organisms also exhibit substantial jurisdictional heterogeneity. This regulatory uncertainty discourages long-term capital investment in novel fermentation technologies.

Fourth, human capital gaps represent a frequently overlooked systemic barrier. Industrial fermentation deployment demands engineering expertise that integrates molecular biology and chemical engineering—a scarce cross-disciplinary skill set [88]. A researcher with deep expertise in molecular biology may lack a foundational understanding of heat transfer and fluid mixing in large bioreactors, while an experienced process engineer may not grasp the mechanistic basis of genetic instability in production strains. Without cross-disciplinary expertise to bridge this knowledge gap, even strains optimized via state-of-the-art DBTL workflows will fail to achieve expected performance on an industrial scale.

Bridging the commercialization valley of death therefore demands more than incremental scientific and technological advances. It requires parallel de-risking of technical, economic, and regulatory dimensions from the earliest stages of process development. In this context, TEA and LCA become indispensable tools—applied not as retrospective evaluation exercises, but as forward-looking design frameworks that guide strain engineering and process development decisions toward long-term industrial viability.

### 4.3. Strategic Recommendations for the Next Decade (to 2035)

Building on the preceding analysis of technical bottlenecks, regional competitive landscapes, and commercialization barriers, five cross-cutting strategic priorities are identified for the global industrial fermentation sector.

First, embed TEA and LCA as Learn phase convergence gates. Quantitative cost (e.g., maximum OPEX per kg) and sustainability (e.g., carbon credit eligibility) thresholds must be defined at the Design phase and treated as non-negotiable convergence criteria at the Learn phase. Any iteration that improves titer but fails to meet pre-defined economic or environmental targets must be returned to Design, regardless of laboratory performance. This shifts TEA/LCA from retrospective justification to forward-acting design constraints. The U.S. Department of Energy’s Agile BioFoundry, for instance, has integrated TEA-LCA workflows that couple process modeling with experimental data to assess economic viability and environmental footprints at each stage of bioprocess development, providing a template for how such gate logic can be operationalized [89].

Second, mandate scale-down simulation as a pre-pilot validation standard. Strain validation must move beyond idealized shake-flask conditions to include miniature bioreactor systems that replicate the spatial gradients, transient fluctuations, and hydrodynamic stresses of production-scale vessels. Commercially available 48-well microbioreactor systems with online monitoring capabilities (e.g., Ambr) enable 80–100 parallel fermentations with intrinsic replicates, while high-throughput microtiter plate (MTP) cultivation combined with automated liquid handling reduces required culture volumes and labor [90,91]. Such platforms directly support the scale-down simulation called for in the Design phase gate. Strains failing to meet performance criteria under these simulated industrial conditions should be cycled back through DBTL for targeted re-engineering before any pilot-scale investment is committed.

Third, establish shared, open-access fermentation data repositories. The limited size, fragmentation, and proprietary nature of industrial datasets remain the primary bottleneck for generalizable AI models. Cross-sector consortia—uniting industry, national labs, and academia—should aggregate anonymized, standardized process data to train robust, cross-platform models, thereby democratizing AI-driven optimization for small- and medium-sized enterprises. The Pistoia Alliance in the pharmaceutical sector exemplifies how pre-competitive data-sharing consortia can operate to overcome industry fragmentation, and similar models for fermentation process data—including proposals for shared repositories with standardized metadata—would significantly improve the training of generalizable AI models [92].

Fourth, harmonize regulatory frameworks across jurisdictions. The current fragmented GMO regulatory patchwork inflates compliance costs and deters investment. International bodies (WHO, FAO, OECD) should advance risk-based, product-centric frameworks that differentiate well-characterized, low-risk engineered strains from those requiring heightened scrutiny. Critically, non-GMO approaches such as adaptive laboratory evolution should receive equivalent regulatory recognition where safety and performance profiles are comparable. Existing platforms such as the OECD’s Working Party on Biotechnology, Nanotechnology and Converging Technologies and the International Pharmaceutical Regulators Program provide institutional frameworks for international dialog that could be leveraged to address regulatory divergence in fermentation products [93].

Fifth, institutionalize cross-disciplinary workforce training. Academic curricula must integrate metabolic engineering, bioprocess engineering, data science, and techno-economic analysis. Industrial stakeholders should implement cross-functional rotation programs to bridge the cultural and technical gap between molecular biologists and process engineers. Developing this hybrid talent pool is a prerequisite for systematically narrowing the genotype–industrial phenotype gap. Existing models include integrated bioengineering–chemical engineering training programs and industry-embedded academic curricula, as well as industrial cross-functional rotation programs and co-location of R&D teams to foster direct, continuous interaction between molecular biologists and process engineers [88].

Collectively, these five pillars operationalize the scale-up-gated DBTL philosophy beyond the laboratory, ensuring that manufacturability, cost competitiveness, and regulatory feasibility are embedded from the earliest design stages rather than addressed as afterthoughts during commercialization.

## 5. Conclusions and Outlook: Toward Fully Autonomous Biomanufacturing

The primary scientific contribution of this review is the formalization of the “scale-up-gated DBTL” framework as a corrective architectural logic for industrial fermentation. Unlike prior reviews that focus on individual tools or specific applications, this work provides a systematic, cross-sectoral diagnostic framework that identifies which decision gate is missing in each industrial context and how that deficit drives scale-up failure. This review has argued that the persistent failure to translate laboratory fermentation innovations into industrial profitability—the so-called valley of death—is not primarily a consequence of inadequate SynBio tools or insufficient AI sophistication. Rather, it is a structural failure of the Design–Build–Test–Learn (DBTL) cycle itself when operated without manufacturability constraints. We have proposed scale-up-gated DBTL as a corrective architecture: a framework in which scale-down simulation constrains the Design phase, downstream processing (DSP) compatibility and industrial robustness metrics gate the Test phase, and TEA with LCA serves as the convergence criterion at the Learn phase. SynBio and AI remain indispensable execution tools, but it is the gates—not the tools—that close the loop and align optimization with factory-floor reality.

Across the three core pillars—strain engineering, process control, and downstream purification—we have shown that unconstrained DBTL systematically converges on laboratory optima that are industrially unviable, whereas workflows that embed sector-appropriate decision gates (Design for energy, Test for pharma, Learn for agriculture) have demonstrated successful pilot-to-commercial transitions. These cases are not exceptions; they are proof of principle that manufacturability, if treated as a design constraint rather than a post hoc verification, can be engineered into the process from the outset.

Nevertheless, scale-up-gated DBTL is necessary but not sufficient. Even a fully gated workflow cannot eliminate barriers that lie outside the loop’s technical logic: high capital expenditure (CAPEX) for greenfield facilities, jurisdiction-specific regulatory fragmentation, and the acute shortage of cross-disciplinary talent capable of bridging molecular biology and chemical engineering. These require parallel institutional and policy interventions—shared data infrastructures, harmonized GMO frameworks, and integrated training curricula—that cannot be delivered by DBTL alone.

Looking ahead to the next decade, we identify three frontier challenges that will define whether gated DBTL evolves into truly autonomous biomanufacturing:

First, the validation bottleneck shifts from biology to computation. As DBTL accelerates strain construction, the rate-limiting step is becoming the time required to validate scale-down models and TEA projections against pilot data. The field must develop predictive scale-down validation platforms that can certify industrial robustness within weeks, not months, using standardized mini-bioreactor arrays coupled with high-frequency offline analytics. Without this, gated DBTL will remain intellectually elegant but practically throttled by downstream validation queues.

Second, AI models must overcome the “silent evidence” bias inherent to fermentation datasets. Industrial failures are rarely published; consequently, models trained on the literature data are systematically over-optimistic about scale-up success. The community must establish open, curated repositories of negative industrial outcomes—failed scale-ups, stability collapses, cost overruns—to train models that recognize failure trajectories, not just success patterns. This is a cultural and institutional challenge as much as a technical one.

Third, dynamic gating requires real-time, intracellular-capable sensors. Current Test-phase gates rely on offline or at-line measurements (HPLC, GC-MS) that report extracellular metabolites with hours-to-days latency. To implement truly closed-loop Learn phase gating, the industry needs molecular biosensors and soft-sensors capable of reporting intracellular pathway flux, redox status, and product localization in real time during scaled-down validation. This would enable the Learn phase to distinguish between “true titer improvement” and “titer improvement achieved by diverting flux that will destabilize DSP performance”—a distinction that current metrics cannot resolve.

In summary, the transition from empirical fermentation craft to autonomous, scale-up-aware biomanufacturing is already underway, and scale-up-gated DBTL provides the operational logic for that transition. But its full realization depends on parallel advances in validation infrastructure, negative-data culture, and real-time sensing. Without these, DBTL—even when gated—remains a laboratory-bound optimization engine. With them, it becomes the blueprint for a resilient, cost-competitive, and sustainable industrial bioeconomy.

## Data Availability

No new data were created or analyzed in this study. Data sharing is not applicable to this article.

## Abbreviations

The following abbreviations are used in this manuscript:

AI	Artificial intelligence
ALE	Adaptive laboratory evolution
ANN–GA	Artificial Neural Network–Genetic Algorithm
CAPEX	Capital Expenditure
CDMO	Contract Development and Manufacturing Organization
cGMP	Current Good Manufacturing Practice
CRISPR	Clustered Regularly Interspaced Short Palindromic Repeats
CRISPRa	CRISPR Activation
CRISPRi	CRISPR Interference
DBTL	Design–Build–Test–Learn
DCS	Distributed Control System
DSP	Downstream processing
*E. coli*	*Escherichia coli*
GMO	Genetically modified organism
GRAS	Generally Recognized as Safe
LCA	Life-cycle assessment
OECD	Organization for Economic Co-operation and Development
QSAR/QSPR	Quantitative Structure–Activity/Property Relationship
SynBio	Synthetic biology
TEA	Techno-economic analysis
